# Isolation and Free Radical Scavenging Ability of Linear Polysaccharides From Cuttlebone of Sepia prashadi

**DOI:** 10.7759/cureus.60163

**Published:** 2024-05-12

**Authors:** Juvairiya Fathima Allapitchai, Annathai Pitchai, Pasiyappazham Ramasamy

**Affiliations:** 1 Physiology, Saveetha Institute of Medical and Technical Sciences, Saveetha University, Chennai, IND; 2 Prosthodontics and Implantology, Saveetha Institute of Medical and Technical Sciences, Saveetha University, Chennai, IND; 3 Polymer Research Laboratory, Centre for Marine and Aquatic Research, Saveetha Institute of Medical and Technical Sciences, Saveetha University, Chennai, IND

**Keywords:** dpph, superoxide radical scavenging assay, innovation, sepia prashadi, chitosan

## Abstract

Background

This study aimed to isolate linear polysaccharides from *Sepia prashadi* cuttlebone with the objective of evaluating their ability to scavenge free radicals. By providing new natural components for pharmaceutical and functional food uses, this research advances our understanding of the potential health benefits of polysaccharides originating from marine sources and their antioxidant properties.

Objective

The objective of the study is to isolate a linear polysaccharide chitosan from *Sepia prashadi* cuttlebone (produced by the partial deacetylation of chitin), characterize its structure using fourier transform infrared spectroscopy (FTIR), scanning electron microscopy (SEM), and X-ray diffraction (XRD), and explore the isolated polysaccharide's free radical scavenging potential.

Material and methods

Linear polysaccharide, chitosan was extracted chemically from *Sepia prashadi* from cuttlebone waste, by demineralization and deproteinization.Chemical characterization of chitosan was performed using Fourier transform infrared spectroscopy (FTIR) in the 400-4000 nm frequency range. The surface characteristics of chitosan, such as its texture, porosity, and roughness, are visible in scanning electron microscopy (SEM) images. X-ray diffraction (XRD) can be utilized to examine how chitosan interacts with other substances, such as medications or nanoparticles, by analyzing alterations in the diffraction pattern during complexation or formulation. Scavenging ability was demonstrated by 2,2-diphenyl-1-picrylhydrazyl (DPPH), superoxide radical, and chelating ability of ferrous ions assays.

Results

Chitosan is formed from chitin. The extraction yields of chitosan and chitin were 78% and 39%, respectively. High levels of superoxide radical scavenging activity (76.1%), DPPH radical scavenging activity (62.1%) and chelating activity (127.5% at 100 g/mL) were observed in cuttlebone chitosan. *Sepia prashadi* showed an increased antioxidant activity in chitosan.

Conclusion

The goal of this study was to determine the effectiveness of various extraction techniques for preserving the antioxidant activity of chitosan derived from *Sepia prashadi* cuttlebone waste. The maximum scavenging activity was demonstrated by both the chelating ability and antioxidant activity. Considering that this raw material is derived from renewable resources and produces highly valued chemicals, it is a profitable endeavor.

## Introduction

Chitin is a structural polysaccharide comprising 1-4 linked N-acetylglucosamine residues. The exoskeletons or cuticles of many invertebrates, including arthropods, molluscs, cnidarians, and pogonophores, as well as the cell walls of algae and fungi, are the primary sources of chitin. Currently, the primary source of industrial chitin is waste from the manufacturing of marine food, primarily the shells of crustaceans, such as krill, shrimp, and crabs [1]. Chitin is a naturally occurring polysaccharide that contains nitrogen and is chemically linked to cellulose poly(1-4)-N-acetyl-d-glucosamine. It is the main component of the exoskeleton or outer layer of insects, crustaceans, and arachnids. Chitin is a somewhat translucent and rigid substance that is generally not soluble in alkaline solutions and organic solvents [2].

Natural polysaccharides are produced by several organisms. Chitin is the most prevalent biopolymer after cellulose, based on the amount generated worldwide each year. Marine crustaceans, such as molluscs, shrimps, crabs, and lobsters, possess distinctive biological characteristics. Polysaccharides, chitin, and calcium constitute the entire exoskeleton of crustaceans. Chitin, which is produced by a variety of organisms including lower plants and animals, is essential for strength and reinforcement. For commercial applications such as medicines, nutraceuticals, cosmetics, and environmental engineering, the yearly output of chitin is projected to be between 2000 and 4000 tons globally [3]. Chitin is insoluble in majority of the solvents, due to its compact structure. To boost the solubility, chitin undergoes chemical changes. From chitin, the most frequent derivative is chitosan, which is obtained through partial deacetylation [1].

Owing to a number of special qualities as low toxicity, biodegradability, and biocompatibility, chitosan has been thoroughly studied for use in several industries. For instance, chitosan has been employed as a dehydrating agent in cosmetics, a medication delivery vehicle, a hydrogel film, an elicitor to activate plant defences, a supplement during food preservation and food additives, and as a flocking agent in water treatment [4]. Chitosan, which includes copper, lead, mercury, and uranium, was first used to chelate dangerous metal ions from wastewater. It is believed that the use of chitosan in humans is safe. Research on the chelating capacity of chitosan began approximately 20 years ago. A study by Muzzarelli et al. showed that chitin, chitosan, and other chelating polymers have been shown to be efficient in chelating transition metal ions. Because of its high amino group content, Muzzaraelli claimed that chitosan was the most effective chelating agent and had the highest collecting ability among all examined polymers [5]. Chitosan has a greater chelation value than a number of other materials, including bark, activated sewage, sludge, and poly amino styrene [6].

Natural antioxidants are generally composed of a wide variety of chemicals including carotenoids, nitrogen compounds, and phenolic compounds. Antioxidants are also present in marine plants and crustaceans, as discovered through habitat studies in the quest for novel antioxidants. The greatest emphasis has been placed on chitosan and its derivatives owing to their evident anticoagulant and antioxidant properties. Furthermore, most studies addressing the antioxidant or antiviral qualities of these compounds, consider the impact of the molecular weight and degree of substitution of sulfated polysaccharides on their biological activity. Thus, efforts have been made to synthesize sulfated chitosan from *S. prashadi* cuttlebone and study its *in vitro* anticoagulant and antioxidant properties [7, 8]. Chitosan has a broad range of inhibitory effects against yeast, molds, Gram-positive and Gram-negative bacteria. It is a nontoxic, nonallergenic, biodegradable polymer with antibacterial action that can be sourced from renewable sources, such as fisheries, and is a major factor in the development of novel uses for this polymer [9].

## Materials and methods

Materials

Sigma Chemical Co. (St. Louis, MO, USA) provided the following products: ascorbic acid, butylated hydroxyanisole (BHA), (2,2-diphenyl-1-picrylhydrazyl), ethylene diamine tetra acetic acid (EDTA), ferrocene, linoleic acid, potassium ferricyanide, and potassium permanganate. Merck Co. (Darmstadt, Germany) provided the hydrogen peroxide (H_2_O_2_) and ferrous chloride (FeCl_2_). All compounds were of analytical grade.

Extraction of chitin and chitosan

Using Takiguchi's approach chitin was recovered from the cuttlebone of *Sepia prashadi* by demineralization and deproteinization [10]. After chitin was extracted from cuttlebone, it was deacetylated using 40% aqueous sodium hydroxide (NaOH) in accordance with Takiguchi's procedure to isolate chitosan [11].

Fourier transform infrared (FTIR) spectral analysis of chitosan

The FTIR spectra of solid samples of chitosan derived from the cuttlebone of *S. prashadi* were acquired using Bruker's Alpha II-FTIR spectrometer (Bruker Corporation, Massachusetts, USA). A standard chitosan sample (Sigma) was also used for comparison.

Scanning electron microscopy (SEM)

The microstructure and surface properties of chitosan were investigated with a scanning electron microscope. A thin film of 40/60 gold/palladium was applied to the sample by directly evaporating the alloy at 20 V using a vacuum evaporator (Hitachi Hus-4, Hitachi Corporation, Japan). An accelerated potential between 0.5 and 30 kV at different magnifications was used for the experiment.

X-ray diffraction (XRD)

The diffraction angle (2θ) and orientation of the specimen were found to be associated with the X-ray diffraction intensity, which was measured using a Shimadzu XRD-6000 device (Shimadzu Corporation, Japan). The crystalline phases of the specimens were identified, their structural characteristics were precisely evaluated, and the size and orientation of the crystallites (small areas of crystallinity) were ascertained using the diffraction pattern.

*In vitro *free radical scavenging assays

DPPH Radical Scavenging Ability

DPPH radical scavenging capacity was assessed using the following methods. One millilitre of a methanolic solution containing DPPH reagent was combined with chitosan (0.1-10 mg/ml) in distilled water to yield a final DPPH concentration of 10 mM/L. After giving the mixture a good shake and allowing it to stand in the dark for 30 min, the absorbance at 517 nm was measured in comparison to a blank. Ascorbic acid was employed as a standard [12].

The scavenging ability was calculated using the following formula:

Scavenging ability (%) = (Abs_517_ of control − Abs_517 _of sample) / Abs_517_ of control * 100

*Superoxide Radical Scavenging Assay* 

The superoxide scavenging ability of chitosan was assessed using the following method. The reaction mixture, containing chitosan polysaccharide in phosphate buffer (0.1 M pH 7.4), was incubated at room temperature for 5 min and the absorbance was read at 560 nm against a blank. α-Tocopherol was employed as control [13].

The capability of scavenging the superoxide radical was calculated using the following equation:

Scavenging ability (%) = (1 − Abs_560_ of sample)/Abs_560_ of control) * 100

Chelating Ability of Ferrous Ions

The ferrous ion-chelating capability of sulfated chitosan was evaluated using the following method. The iron-containing ferrozine complex was measured at 562 nm to measure the sulfated chitosan's Fe^+2^ chelating capacity. In summary, the reaction mixture was diluted to a total volume of 0.8 ml with water, agitated thoroughly, and incubated for 10 min at room temperature. It contained various concentrations of chitosan (CS), FeCl_2_ (2 mM), and ferrozine (5 mM). The absorbance of the mixture was measured relative to a blank at 562 nm. EDTA was used as the positive control [14].

The following formula was used to determine the capacity of chitosan to chelate ferrous ions:

Chelating effect (%) = (1- Abs_562_ of sample)/ Abs_562_ of control) * 100

## Results

Yield of chitin and chitosan, FTIR spectral analysis

**Figure 1:**
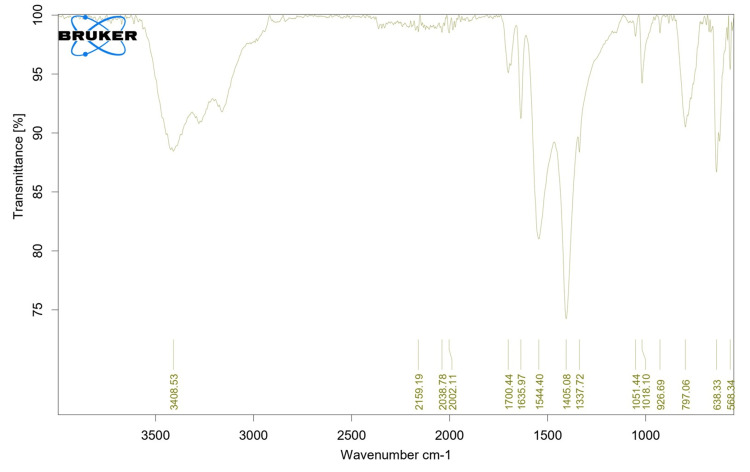
Fourier transform infrared spectral analysis (FTIR) of linear polysaccharide from Sepia prashadi

The chitosan and chitin yields were 78% and 39%, respectively. The infrared spectra of the sample were recorded in the 400-4000 cm^−1^ wave number range. The FTIR spectrum of chitosan revealed significant absorption peaks suggestive of several functional groups; these peaks correlated with the C-F stretching vibrations typical of fluoro compounds. Moreover, discrete, highly intense peaks at 1051 cm^−1^ indicate C-O stretching in the main alcohol. In addition to alcohol, a significant absorption at 1635 cm^−1^ indicated the presence of the secondary amide complex C=O stretching (Figure 1). A significant absorption at 2038 cm^−1^ indicates the N=C=S stretching of an isothiocyanate molecule. A signal with medium strength at 2159 cm^−1^ suggests the C=C stretching of an alkene molecule.

Scanning electron microscope (SEM)

**Figure 2:**
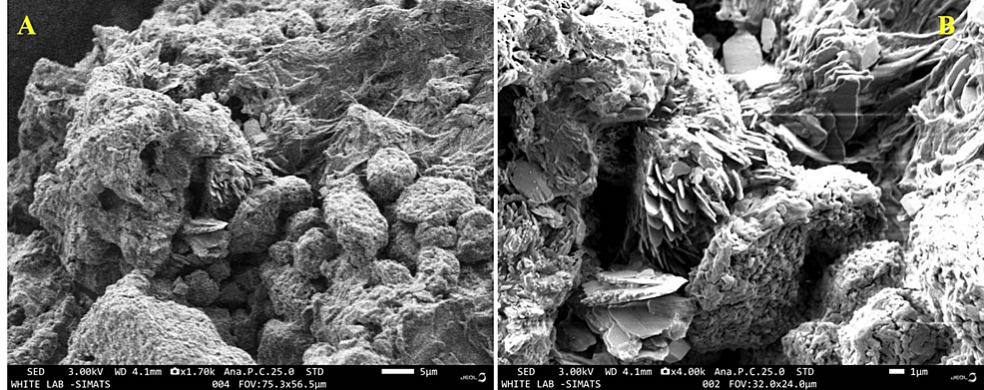
Scanning electron microscopy (SEM) image of linear polysaccharide from Sepia prashadi (A, B)

The scanning electron microscope (SEM) image displays a random arrangement of grains, providing a cross-sectional view. Additionally, captured image of chitosan resembled the upper surface of a piece of bread. The near-spherical form of chitosan, which might be useful in biological applications, was further supported by SEM images (Figures 2A, 2B).

X-ray diffraction (XRD)

**Figure 3:**
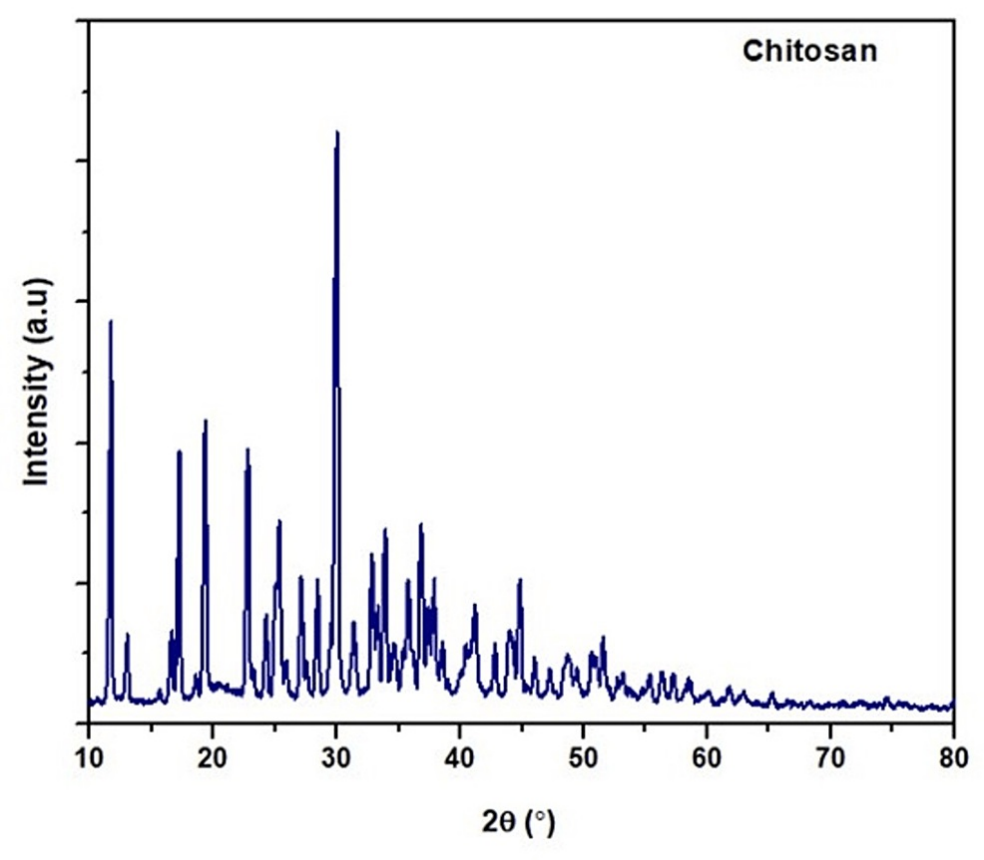
X-ray diffraction of linear polysaccharide from Sepia prashadi

Chitosan yielded 50 strong peaks in all, with the largest peak occurring at 2θ 30.02° (5674 counts/s) and sharp peaks observed between 11-30.02° (2159-5674 counts/s) (Figure 3).

In vitro free radical scavenging assays

DPPH Radical Scavenging Activity

**Figure 4:**
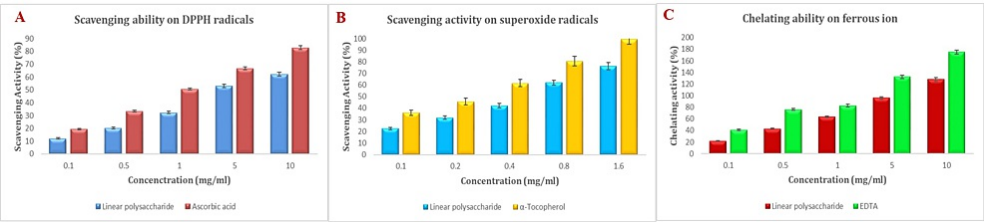
Free radical scavenging ability of linear polysaccharide from Sepia prashadi: A) DPPH radical scavenging activity, B) Superoxide radical activity, C) Chelating ability of ferrous ions

Figure 4A illustrates how chitosan and ascorbic acid scavenge DPPH radicals. Chitosan had a strong scavenging ability of 62.17% at 10 mg/ml. The 50% inhibitory concentration (IC_50_) for DPPH radical scavenging was determined to be 4.53 mg/ml. Ascorbic acid exhibited a DPPH radical scavenging activity of 82.78%.

Superoxide Radical Activity

The superoxide radical scavenging activity was significantly inhibited by chitosan, and this effect was concentration dependent. At all tested chitosan doses, a considerable super radical scavenging effect (range of 22.3 to 76.1%) was observed. Figure 4B shows the impact of chitosan on the oxidative damage caused by Fe^+3^/H_2_O_2_ on deoxyribose. At the highest dose, approximately 76.1% inhibition was detected, whereas 99% inhibition was reported for α-tocopherol.

Chelating Ability of Ferrous Ions

Chitosan exhibited 127% chelating activity towards ferrous ions. However, EDTA exhibited a chelating capacity of 174% (Figure 4C).

## Discussion

Exoskeletons of crustaceans, insects, and fungal cell walls are the main sources of chitin, which is the second most common biopolymer in the natural world. Chitin is converted to chitosan by deacetylation, usually with alkaline hydrolysis. Various studies have reported differences in chitin production among different species. For example, the reported chitin yields vary greatly between species: 20% in *Sepia officinali*s cuttlebone and 36.06%, 36.55%, and 22.18% in *Loligo lessoniana*, *Loligo formosana*, and *Penaeus monodon*, respectively [15]. In the current study, we discovered that 61% of chitin in the *Sepia prashadi* cuttlebone was extracted. Notably, chitin is insoluble in water even though it is a polymer, which restricts its application. Numerous scientists have created chitin derivatives, with chitosan being a well-known derivative, in an effort to improve its solubility and broaden its use. Karthik et al. determined that the chitosan output from the squid *Doryteuthis sibogae* gladius was 33.02% and from the shell and operculum of *Nerita crepidularia* was 35.43% [16]. Chitosan, the deacetylated form of chitin, acts as a building block for other changes. Compared to the yield of chitosan from *S. pharaonis* and *Donax scortum*, the yield percentage of chitosan was relatively greater [17].

The FTIR spectra of chitosan usually exhibit distinctive peaks linked to their functional groups. For instance, a peak at approximately 3400-3500 cm^-1^, which corresponds to the stretching vibration of N-H bonds, indicates the presence of an amine group (-NH_2_). In chitosan, the carbonyl stretching vibration (C=O) of the amide group (CONH) is represented by a band centered at approximately 1650-1655 cm-1 [18]. Usually, the stretching vibrations of the C-O-C glycosidic bonds in the polysaccharide backbone of chitosan are detected at approximately 1050-1150 cm^-1^. Furthermore, the bending vibrations of the C-O and C-N bonds are reflected as peaks in the 890-1150 cm^-1^ area, which offers further structural details regarding chitosan [11]. The vibrational shift of chitosan was detected at 474 and 2433 cm^-1^, indicating the presence of chitosan. Raman peaks at 1658 cm^-1^ were produced by extracted chitosan.

Chitosan can be used to create a variety of products including films, microspheres, and nanoparticles. The size, shape, and distribution of the particles inside these formations were characterized using SEM. This information is essential for streamlining the manufacturing procedures and customizing chitosan materials for intended use. It is possible to compare various chitosan formulations, processing methods, or alterations using SEM imaging. Through visual analysis of variations in the surface shape and structure, scientists may determine which chitosan materials are most suited for certain uses or enhance existing formulations. The SEM image of chitosan displays an intriguing microstructure composed of a web of interwoven fibers or particles. The surface seems comparatively smooth, with holes and sporadic abnormalities all over it.

The chitosan fibers and particles showed a range of diameters, suggesting possible differences in the production process or the existence of aggregates. It is challenging to compare earlier research because the specimens were obtained from various sources and locations, and they were also processed for SEM imaging using a wide range of techniques [19]. Chitosan usually shows diffraction peaks in its XRD pattern, which correlate with its crystalline areas. The strength and position of these peaks provided information about the molecular packing and order inside the chitosan structure. In the present study, we found that the chitosan showed similar results to those reported by Rasti et al. [20]. They observed clear and distinct reflections for chitosan derived from mollusc chitin within the temperature range of 30 to 35° [20]. Chitosan is found in the exoskeletons of crustaceans, has hydroxyl and amino groups in its chemical structure, and is naturally an antioxidant. Owing to these functional groups, chitosan can reduce oxidative damage in biological systems by scavenging free radicals.

The test known as DPPH (2,2-diphenyl-1-picrylhydrazyl) is frequently employed to assess the antioxidant capacity of various substances such as chitosan. Numerous studies have examined the ability of chitosan and its derivatives to scavenge DPPH, suggesting that they may have antioxidant properties. Because chitosan's amino groups may provide electrons to neutralize free radicals, it can scavenge DPPH radicals. Our investigation revealed that *Sepia prashadi* has a 62.17% scavenging activity. In comparison, the scavenging capacity of chitosan from *S. lessoniana* was 55.48% at 10 mg/ml [21], whereas the scavenging ability of crab chitosan C60 on DPPH radicals was 28.4% at 10 mg/ml [22].

Superoxide radicals (O^-2^) are reactive oxygen species produced naturally during cellular metabolism. They are implicated in several oxidative stress-related illnesses, including cancer, neurological conditions, and cardiovascular illnesses. Superoxide radical scavenging tests measure the capacity of a compound to neutralize or quench damaging radicals to assess its antioxidant activity. Significant superoxide radical scavenging activity was observed at all chitosan concentrations tested. The percentages of chitosan from *Sepia prashadi *and α-Tocopherol that were able to scavenge superoxide radicals at a concentration of 0.5 mg/ml were 42.17%, 61.92%, and 76.19%, respectively [23]. One fascinating property of chitosan chemistry is its ability to chelate substances. This property has important applications in environmental research, agriculture, medicine, and manufacturing, among other fields. Chitosan polysaccharide, which is produced from chitin, has excellent chelating qualities because of its amino groups, which may bind to metal ions. Because of this property, chitosan has a wide range of applications. At 10 mg/ml, the chelating activity of chitosan derived from *Sepia prashadi* on ferrous ions was 127.59% in the current study. At 10 mg/ml, EDTA exhibited a strong chelating ability of 174.92%. At a concentration of 1 mg/ml, fungal chitosans exhibit full chelating capacity [24].

Limitations

The antioxidant test offers insightful data on the ability of chitosan to scavenge, but it is crucial to keep in mind that the antioxidant activity observed in this assay could not accurately reflect the antioxidant behavior of chitosan in intricate biological systems. The overall antioxidant capabilities of chitosan are also influenced by other variables, including its particular modes of action, interaction with other chemicals, and bioavailability. Ultimately, more research employing structural characterization techniques such as nuclear magnetic resonance (NMR) and in vivo models is required to completely comprehend the potential health advantages of chitosan and its use as an antioxidant, even though the DPPH assay can provide light on the antioxidant activity of the material.

## Conclusions

In summary, the present study effectively isolated chitosan from the cuttlebones of *S. prashadi*. Additionally, this study provides opportunities to investigate the antioxidant properties of chitosan prepared from the cuttlebone of *S. prashadi*. Because of its special qualities, chitosan has demonstrated promise in a number of applications, and its potential as an antioxidant is an intriguing subject for further research. Comprehending the antioxidant properties of chitosan can have consequences for industries such as medicine and food preservation. The correlation between the superoxide radical scavenging test and chitosan highlights the potential of chitosan as a natural antioxidant with intriguing applications in healthcare and biological research. Novel therapeutic approaches may be enabled by further research into the antioxidant processes of chitosan and its interaction with biological systems.
